# Resolution of Q Fever–Associated Cryoglobulinemia With Anti-CD20 Monoclonal Antibody Treatment

**DOI:** 10.1177/2324709616686612

**Published:** 2017-01-01

**Authors:** Kellie L. Hawkins, Edward N. Janoff, Robert W. Janson

**Affiliations:** 1 University of Colorado, Aurora, CO, USA; 2 Denver Veterans Affairs Medical Center, Denver, CO, USA

**Keywords:** Q fever, cryoglobulinemia, endocarditis, rituximab

## Abstract

Immunologic phenomena can complicate chronic infections with *Coxiella burnetii* (Q fever), including immune complex deposition causing vasculitis, neuropathy, and glomerulonephritis. We describe the case of a man with Q fever endocarditis, mixed cryoglobulinemia, and life-threatening vasculitis driven by immune complex deposition who was successfully treated with B cell depleting therapy (rituximab).

## Case Report

A 71-year-old male was admitted for evaluation of night sweats, weight loss, and a vasculitic appearing rash. Six months prior to admission, he developed shortness of breath and lower extremity edema. Soon thereafter, he developed palpable purpura on his upper and lower extremities, with skin biopsy showing leukocytoclastic vasculitis (LCV). The rash improved in part with prednisone prescribed by his primary care physician; however, drenching night sweats and shortness of breath continued. He lost approximately 30 pounds over 6 months. An outpatient transthoracic echocardiogram showed a heavily calcified and thickened mitral valve with moderate mitral regurgitation, moderate mitral stenosis, and severe pulmonary hypertension. He was referred for rapid mitral valve replacement.

Past medical history included gout, dermatomal herpes zoster, benign prostatic hypertrophy, osteoarthritis, and rheumatic fever as a child, without prior known valvular abnormality. Outpatient medications included combination ipratropium bromide/albuterol sulfate inhaler, ASA 81 mg, and prednisone 10 mg BID (for the rash). Allergies included pruritus with allopurinol. Family history was noncontributory.

The patient was an antique dealer from a small town in Montana. His only foreign travel was to Korea while in the Army. His dog was recently sick after playing with cow and elk bones that neighbors threw into his yard. He carved knifes from African ivory and created jewelry with bear claws and exotic animal skins (eg, zebra). He did not use tobacco or illicit drugs, and he drank 2 to 3 beers per week.

**Figure 1.**
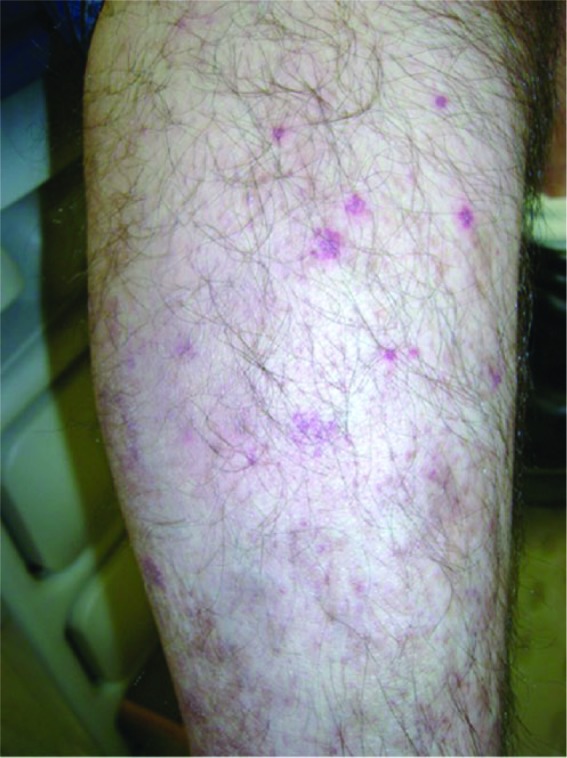
Course of patient with Q fever endocarditis and cryoglobulinemia.

On admission, vital signs were normal, but he appeared chronically ill. Pertinent positives on exam included holosystolic and diastolic murmurs. Skin showed palpable purpura on his bilateral lower extremities (Figure 1).

**Figure 2.**
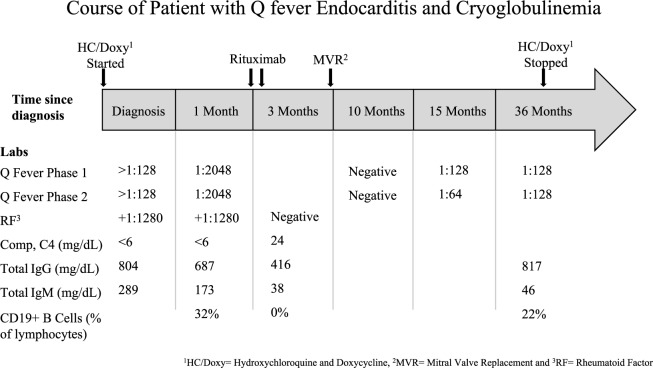
Representative picture: right lower extremity palpable purpura, provided courtesy of R. W. Janson.

Extensive blood work (summarized in Figure 2) was notable for a positive rheumatoid factor (RF) + 1:1280 (reference range <1:80) and C4 at <6 mg/dL (reference range 20-59). Serum creatinine was 1.1 mg/dL (reference range 0.6-1.3). Anticardiolipin IgG was negative but IgM was positive. C-reactive protein level was 123 mg/L (reference range <2.99), erythrocyte sedimentation rate was 75 mm/h (reference range 0-13). Cryoglobulins, hepatitis C IgG, HIV-1, ANCAs, antinuclear antibodies (ANA), and *Bartonella* and *Brucella* serologies were negative. The positive RF and low C4 prompted an evaluation for causes of cryoglobulinemia. IgG phase 1 and phase 2 titers for *C burnetii* returned positive at >1:128, measured by indirect fluorescent antibody assays (titers were not further diluted at that time). Repeat biopsy of the rash confirmed LCV. He was given a diagnosis of Q fever endocarditis complicated by LCV. The initial source of *C burnetii* may have been the patient’s dog.

Prednisone was uptitrated (60 mg daily), and he was started on doxycycline (100 mg BID) and hydroxychloroquine (200 mg TID) with a planned 12- to 18-month course. Mitral valve replacement surgery was deferred 1 month until prednisone could be tapered due to concerns about poor wound healing while on high doses of prednisone. However, on the day of planned surgery, he experienced a lower gastrointestinal bleed, new-onset neuropathy (left foot drop), and worsening of LCV rash.

Colonoscopy showed ulcerated mucosa from the cecum to the transverse colon. Biopsies were consistent with ischemic colitis. RF remained elevated at 1:1280 (reference range <1:80), C4 remained low at <6 mg/dL (reference range 20-59), and acute kidney injury was detected (peak creatinine 1.7 [reference range 0.6-1.3]). Type II cryoglobulins were now detected in the plasma. Repeat *C burnetii* IgG phase 1 and phase 2 titers were both 1:2048, and ribosomal DNA for this organism was detected in the serum by polymerase chain reaction. The possibility of embolic disease as the cause of his ischemic colitis was entertained, but given his clinical picture we postulated that his rash, neuropathy, and ischemic colitis resulted from immune complex deposition in the setting of a mixed cryoglobulinemia with Q fever endocarditis.

The patient was restarted on prednisone 60 mg/day for treatment of type II mixed cryoglobulinemia, and mitral valve surgery was again delayed due to the potential effects of steroids on sternal wound healing.^1^ Alternatives to prednisone were explored given the rapid and systemic involvement of his type II cryoglobulinemia and supposition that immune complex production would be ongoing until the mitral valve could be replaced. Rituximab (Biogen and Genentech; Cambridge, MA, and San Francisco, CA, respectively) was considered as it had been used successfully to treat cryoglobulinemia during hepatitis C infection but not with Q fever.^2^

Two doses of rituximab 1000 mg intravenously were administered 14 days apart. His rash resolved within 7 days of the first dose of rituximab; repeat colonoscopy showed healing mucosa, his neuropathy and drop foot improved, and prednisone was tapered from 60 mg/day to 5 mg/day over 6 weeks. Laboratory results normalized with a negative RF, normal C4, normal inflammatory markers, and normal renal function. However, IgG phase 1 and phase 2 *C burnetii* titers remained elevated at 1:2048. His symptoms and laboratory parameters improved after rituximab therapy, and he successfully underwent mitral valve replacement 2 months after his last dose of rituximab. Prior to surgery, he received intravenous immunoglobulin (400 mg/kg × 1) for a low serum IgG of 416 mg/dL. Culture of the valve tissue was negative, but polymerase chain reaction detected *C burnetii* 16S ribosomal DNA. Prednisone was tapered over 8 weeks and discontinued. The patient remained free of cardiac, dermatologic, and neurologic symptoms over the subsequent 36 months of therapy with oral doxycycline 100 mg BID and hydroxychloroquine 200 mg TID. After 36 months, IgG phase 1 and phase 2 Q fever titers had declined to 1:128 and therapy was stopped (Figure 2). The decision to treat for 36 months was based largely on difficulties with follow-up and attainment of laboratory data as the patient lived in a remote area and was tolerating doxycycline and hydroxychloroquine with no side effects.

## Discussion

We report the resolution of persistent vasculitis and multi-organ sequelae resulting from mixed cryoglobulinemia with Q fever endocarditis following B cell–depletion therapy. Only 1% to 5% of all acute *C burnetii* infections progress to chronic disease.^3^ Chronic Q fever can manifest with nonspecific constitutional symptoms, such as fever, drenching night sweats, and weight loss. Endocarditis occurs in up to 75% of chronic Q fever cases, and as with this case, a predisposing valvular abnormality is associated with an increased risk of developing Q fever endocarditis.^4^ The incidence of endocarditis in the presence of preexisting valvular pathology is estimated to be nearly 40%.^4^

In Q fever endocarditis, valvular vegetations are often small and require a trained echocardiographist to detect, so the diagnosis should be considered with preexisting valvulopathy and fever of unknown origin. Cutaneous vasculitis has been described with Q fever endocarditis.^5^ Rheumatoid factor and antinuclear antibodies are detectable in 60% and 35% of chronic Q fever cases, respectively.^5^ The presence of antiphospholipid antibodies, including anticardiolipin, at high levels appear premonitory for endocarditis, occurring nearly 25 times more commonly in those who develop this sequela.^6^

Both type II mixed cryoglobulinemia and Q fever endocarditis can manifest with fever, rash, low C4, and glomerulonephritis.^7^ In contrast to the patient described above, the 6 other case reports of Q fever endocarditis associated with mixed cryoglobulinemia had prosthetic valves.^7^ To our knowledge, our patient is the first to have Q fever endocarditis of a native valve associated with mixed cryoglobulin disease. Type II mixed cryoglobulinemia causes vasculitis by deposition of circulating immune-complexes, specifically cryoglobulins that contain a polyclonal IgG and a monoclonal IgM with RF activity.^8^ Among patients with hepatitis C virus (HCV) infection and type II mixed cryoglobulinemia, up to 83% show skin manifestations.^9^ Other less common features of type II mixed cryoglobulinemia, specifically in HCV-negative individuals, include peripheral neuropathy (52% of cases), kidney involvement (35% of cases), and gastrointestinal involvement (5% of cases).^9^ The patient described in this case had cutaneous manifestation of cryoglobulins but also had gastrointestinal bleeding, peripheral neuropathy, and probable glomerulonephritis.

The initial management of mixed cryoglobulinemia, of which HCV-related cryoglobulinemia is the most common, is to treat the underlying disease. In this case, the Q fever was the antigenic source driving this patient’s cryoglobulin-induced vasculitis and initial management was focused on treating the Q fever with doxycycline and hydroxychloroquine. Rapidly progressive disease, including organ- or life-threatening disease, merits immunosuppressive therapy regardless of the underlying cause of cryoglobulins. Glucocorticoids and cyclophosphamide have been used in the past with varying success.^10^ In this case, the vasculitis and other complications necessitated immunosuppressive therapy. The patient required prompt replacement of his failing mitral valve for both symptom and source control. Surgical repair would have been compromised by a prolonged course of steroids.

B-cell depletion therapy with the humanized chimeric anti-CD20 monoclonal antibody rituximab is used to treat B cell malignancies, autoimmune disorders, and organ transplant rejection. Rituximab has been used with and without steroids for treatment of severe mixed cryoglublinemia.^9^ In the setting of HCV infection, responses rates were more favorable with rituximab versus traditional immunosuppressive therapy (cyclophosphamide and azathioprine) at 12 (64% vs 4%) and 24 months (61% vs 4%).^11^ In addition, those who fail traditional immunosuppressive therapy^8^ and anti-HCV treatment^10^ may also respond to rituximab, including patients with associated neuropathy^12^ and glomerulonephritis. Rituximab and steroids also appear to be more effective than steroids alone for mixed cryoglobulinemia secondary to other disease entities.^9^

In summary, the patient described in this case had multiple signs of severe mixed cryoglobulin disease despite antimicrobial treatment directed at *C burnetii.* The use of high-dose glucocorticoids imposed an unacceptable risk for mitral valve replacement, the definitive treatment for his Q fever endocarditis, and his resulting cryoglobulin-associated vasculitis. Following rituximab therapy, glucocorticoids were rapidly tapered, and he successfully underwent mitral valve replacement 8 weeks after rituximab was initiated, with full resolution of all symptoms. Thus, we report the first case of resolution of *C burnetii*–associated cryoglobulinemia, vasculitis, and neuropathy with rituximab, which permitted successful definitive source control with valvular surgery.
